# Imaging Modalities for Cervical Spondylotic Stenosis and Myelopathy

**DOI:** 10.1155/2012/908324

**Published:** 2011-07-20

**Authors:** C. Green, J. Butler, S. Eustace, A. Poynton, J. M. O'Byrne

**Affiliations:** ^1^Department of Trauma & Orthopaedic Surgery, Royal College of Surgeons in Ireland, Cappagh National Orthopaedic Hospital, Finglas, Dublin 11, Ireland; ^2^Mater Misericordiae University Hospital, Eccles Street, Dublin 7, Ireland

## Abstract

Cervical spondylosis is a spectrum of pathology presenting as neck pain, radiculopathy, and myelopathy or all in combination. Diagnostic imaging is essential to diagnosis and preoperative planning. We discuss the modalities of imaging in common practice. We examine the use of imaging to differentiate among central, subarticular, and lateral stenosis and in the assessment of myelopathy.

## 1. Introduction

Imaging modalities for cervical spondylosis aim to assist the clinician in differentiating discogenic neck pain, radiculopathy, and myelopathy. Radiological assessment helps to localise the site and level of the disease for preoperative planning when surgical intervention is required. The current modalities in common use are pain film roentgenology, magnetic resonance imaging, and computed tomography.

Despite advances in diagnostic imaging plain film remains an inexpensive initial radiological evaluation of the spine in cervical spondylosis. Anteroposterior, lateral, and oblique radiographs can be acquired easily at the time of consultation. These images can show changes in the facet and uncovertebral, osteophytes, and disc space [1]. This is an indication of the underlying pathology but not diagnostic as these findings are common in the adult population [1]. Weight-bearing plain films can also assess alignment and sagittal canal diameter. Measurement of the anteroposterior diameter is typically determined on a lateral plain film as the distance from the posterior surface of the vertebral body to the closest point on the spinolaminar line at the pedicle level. However, this is a two-dimensional assessment of a three-dimensional structure and such measurements have shown to be inaccurate. Three-dimensional imaging modalities are now used for more accurate assessment. Lateral flexion-extention views are also useful initial investigations [2]. These will help to assess cervical range of motion and identify fused segments and instability. Instability is suggested where translation of >3.5 mm and sagittal plane angulation of >11 degrees are present [3].

Compared with other radiological studies available to evaluate the spine magnetic resonance imaging (MRI) provides the greatest range of information [4]. It provides an accurate morphological assessment of both osseous and soft tissue structures including intervertebral discs, spinal ligaments, and the neural elements. Dynamic weight bearing MRI has recently been championed as the preferred technique for pathology-specific diagnosis [5, 6]. Computed tomography in isolation lacks the soft tissue detail achieved with MRI scanning. However, CT is still a useful modality when there is a contraindication to MRI and where metal artefact is obstructing the anatomy. CT myelography is an invasive procedure and is associated with a number of risks. It is only used for patients who have contraindications, equivocal findings, or failed MR imaging because of metal artefact. 

Imaging for spinal stenosis should aim to determine the site of compression. Spinal stenosis can be divided into central, subarticular recess (lateral recess), and lateral [7]. Central stenosis results in concentric narrowing of the spinal canal and can result in cervical myelopathy. Radicular symptoms can be attributed to either subarticular recess stenosis in lateral aspect of the central spinal canal or lateral stenosis at the foramina. Radiological evaluation of the spinal cervical spine can as such be broadly slit into central and lateral.

## 2. Central Radiological Assessment: Central Stenosis and Myelopathy

Modalities employed for a central assessment of the cervical spine should determine the extent and site of canal stenosis and any associated myelopathy.

## 3. Assessment of Sagittal Diameter of the Spinal Canal

The size of the cervical spinal canal is clinically important [4, 8]. The spinal canal is narrowed with central stenosis, and this can lead to cervical myelopathy. The role of the narrow cervical spine in the expression of clinical syndromes was evaluated by Edwards and LaRocca [9]. They predicted that patients with a canal size of <10 mm had myelopathy, those with a canal size of 13 to 17 mm were less prone to myelopathy but were more prone to symptomatic cervical spondylosis, and those with a canal size of greater than 17 mm were asymptomatic [9]. MRI studies which take into account soft tissue structures, weight-bearing, and dynamic imaging have suggested that a congenital sagittal diameter of <13 mm is a significant risk factor for development of stenosis [4]. However, a number of authors have reported an incidence of asymptomatic stenosis of between 16 and 19% [10, 11]. With MRI scanning becoming more routinely available the best management of this group of individuals will be challenging.

There are numerous ways to evaluate the diameter of the spinal canal. Although traditionally determined on a lateral plain film such measurements have shown to be inaccurate. Inaccuracy has also been attributed to variation in the distance from the X-ray source and rotation of the subject [4, 8]. In order to improve accuracy of this measurement on plain film a number of authors have described the use of a ratio between the sagittal diameter of the vertebral body and the diameter of the canal [12, 13]. Pavlov's ratio was considered normal when >1 and stenotic when <0.8. However, some authors have reported a poor correlation between the space available for the cord and the Pavlov ratio [14, 15]. 

The most accurate measurement of spinal canal diameter is obtained using MRI. Unlike other modalities MRI takes into account both osseous and soft tissue structures when calculating the canal diameter. This is important as central stenosis is often due to a combination of degenerative hypertrophy of the facet joints, osteophytic spurring, ligamentum flavum thickening, ossification of the posterior longitudinal ligament, posterior disc protrusion, and translation of one anatomical segment on the next [7]. The examination should be performed using thin sections and high resolution. Spinal MRI should include imaging sets obtained in the axial and sagittal planes using T1-weighted, proton-density, and T2-weighted techniques. In addition pulse sequences that provide high signal from cerebrospinal fluid (myelographic effect) help delineate epidural pathological processes such as disc fragments and osteophytes [16]. The bony and osteophytic components of the spinal stenosis pattern are seen best using a T2-weighted gradient-echo technique.

## 4. Myelopathy

As well as the anatomy of spinal cord compression MRI can show the pathological spinal cord changes in cervical spondylotic myelopathy. Signal change not only indicates the presence of myelopathic change but has also been used as a predictor of outcome [17]. Takahashi et al. were the first group to correlate a high signal on T2-weighted MR images with a poor clinical result after both operative and nonoperative management [18]. However, controversy exists in the interpretation of signal changes in the spinal cord. This may explain why although some studies have shown similar results to Takahashi et al. other studies have not [19, 20]. 

Myelopathy is seen as increased signal within the cord on T2-weighted and a decreased signal on T1-weighted MRI. However, these signal changes are not reciprocal and are likely to represent different underlying pathology [21]. Attempts have been made to correlate MRI and histological findings. Oedema and gliosis have been described as a high-intensity signal change on T2-weighted MRI, and myelomalacia and necrosis as a low-intensity signal change on T1-weighted MRI [22]. This is an important distinction as it suggests that those changes seen with increased intensity on T2 images are reversible whereas those seen a low signal on T1 are irreversible. However, other authors suggest that all increased signals in the spinal cord represent diffuse neuronal cell loss, replacement by glial cells in the stroma, and axonal and spongy degeneration in the white matter indicating advanced spinal cord damage [23]. Radiological classifications systems to quantify changes in signal intensity have been developed in an attempt to identify the radiological divide between reversible and irreversible changes [24]. The simplest of these describes three grades absent, obscure, and bright [25]. But a more detailed classification system that accommodates both T1- and T2-weighted MRI is more predictive of surgical outcome than those that include T2-weighted changes alone [17]. In addition, postoperative MRI has been used to identify late onset of low T1 SI changes in patients with poor neurological recovery [17].

It seems intuitive that multisegmental increased signal change on T2-weighted images would indicate a more severe and extensive pathology and be associated with poor clinical course. However, despite studies showing that multisegemntal disease is associated with a poor functional recovery [26] and more extensive pathology [19] others have shown a mild cervical myelopathy in patients with extensive high signal change [17, 27].

## 5. Lateral Radiological Assessment: Radiculopathy

Radicular symptoms can be attributed to either subarticular recess stenosis in lateral aspect of the central spinal canal or lateral stenosis at the foramina. Detailed history and examination findings are essential to interpreting the results of these scans. The distribution of radiculopathy should be localised to a nerve root. Imaging should be used to ascertain if compression of that nerve root is occurring. Where impingement is demonstrated and surgery is being considered the exact location of obstruction needs to be identified. Preoperative planning should distinguish between subarticular recess stenosis at the same level as the exiting nerve root and lateral stenosis at the foramina below. 

As discussed MRI is the diagnostic standard for evaluation of the cervical spine. However, exaggeration of foraminal stenosis is associated with gradient-echo axial MR imaging scans obtained through the cervical region [28]. Foraminal stenosis has been reported in as high as twenty percent of asymptomatic subjects older than forty years of age [10]. As a result some surgeons carry out a CT myelogram preoperatively. Compressive osteophytes and foraminal stenosis are best identified with use of CT scans [2]. CT myelography has been reported superior to MRI in distinguishing osseous from soft tissue compression of neural structures at the foramina [29, 30]. However, due to the well-documented rick factures associated with cervical myelopathy this examination should be reserved for specific circumstances where MRI will not suffice.

## 6. Future Techniques

Intraoperative ultrasound has been described to be useful during central corpectomy for compressive cervical myelopathy. It is inexpensive and simple imaging modality. It is helpful in identifying the vertebral artery and the trajectory of approach [31]. However, ossification of the posterior longitudinal ligament limits the use of this technique [31]. Development of advanced MRI techniques such as diffusion tensor imaging has shown promise in intramedullary microarchitectural analysis with improved imaging quality and increased lesion identification when compared to conventional MRI [32]. Metabolic neuroimaging has been described for image acquisition from the spinal cord. Findings on high-resolution 18F-fluorodeoxyglucose positron emission tomography (FDG-PET) have been compared with clinical scores and findings on magnetic resonance imaging in patients undergoing surgery for myelopathy [33]. FDG-PET findings correlated with preoperative scores, postoperative scores, and the rate of postoperative improvement, but they had no correlation with high-intensity intramedullary signal changes on T2-weighted images. The major limitation of this technology is the poor resolution of PET scans. Future technological advancements in PET scanning may facilitate evaluation of early spinal cord damage and provide indications for surgical intervention.
